# Identification of a genomic cluster related to hypersecretion of intestinal mucus and mucinolytic activity of atypical enteropathogenic *Escherichia coli* (aEPEC)

**DOI:** 10.3389/fcimb.2024.1393369

**Published:** 2024-12-04

**Authors:** Liana de Oliveira Trovão, Mônica Aparecida Midolli Vieira, Ana Carolina de Mello Santos, Juan Josue Puño-Sarmiento, Pedro Henrique Soares Nunes, Fernanda Fernandes Santos, Victória Galdino Pavlenco Rocha, Terezinha Knöbl, Fernando Navarro-Garcia, Tânia Aparecida Tardelli Gomes

**Affiliations:** ^1^ Laboratório Experimental de Patogenicidade de Enterobactérias, Departamento de Microbiologia, Imunologia e Parasitologia, Escola Paulista de Medicina, Universidade Federal de São Paulo, São Paulo, Brazil; ^2^ Laboratório Alerta, Departamento de Medicina, Escola Paulista de Medicina, Universidade Federal de São Paulo, São Paulo, Brazil; ^3^ Departamento de Patologia, Faculdade de Medicina Veterinária e Zootecnia, Universidade de São Paulo, São Paulo, Brazil; ^4^ Departamento de Biología Celular, Centro de Investigación y de Estudios Avanzados del IPN (CINVESTAV-IPN), Mexico City, Mexico

**Keywords:** atypical EPEC, mucus hypersecretion, mucinases, genomic analysis, virulence factors, rabbit ileal loop

## Abstract

Enteropathogenic *Escherichia coli* (EPEC) strains are subdivided into typical (tEPEC) and atypical (aEPEC) according to the presence or absence of a virulence-associated plasmid called pEAF. Our research group has previously demonstrated that two aEPEC strains, 0421-1 and 3991-1, induce an increase in mucus production in a rabbit ileal loop model *in vivo*. This phenomenon was not observed with a tEPEC prototype strain. Few studies on aEPEC strains evaluating their capacity to induce intestinal mucus hypersecretion were done. This study aimed to investigate aEPEC strains regarding their genotypic and phenotypic characteristics, their ability to alter mucus production in an *in vivo* intestinal infection model, and their potential mucinolytic activity. To investigate the relationship between strains 0421-1 and 3991-1 and 11 other aEPEC strains, their serotypes, sequence types (ST), and virulence factors (VF), several sequencing and genomic analyses were carried out. The study also involved researching the reproduction of mucus hypersecretion in rabbits *in vivo*. We found that the two mucus-inducing strains and two other strains (1582-4 and 2531-13) shared the same phylogroup (A), ST (378), serotype (O101/O162:H33), and intimin subtype (ι2), were phylogenetically related, and induced mucus hypersecretion *in vivo*. A wide diversity of VFs was found among the strains, confirming their genomic heterogeneity. However, among the genes studied, no unique virulence factor or gene set was identified exclusively in the mucus-inducing strains, suggesting the multifactorial nature of this phenomenon. The two strains (1582-4 and 2531-13) closely related to the two aEPEC strains that induced mucus production *in vivo* also induced the phenomenon. The investigation of the mucinolytic activity revealed that all aEPEC strains used mucins as their carbon sources. Ten of the 13 aEPEC strains could cross a mucin layer, and only four adhered better to agar containing mucin than to agar without mucin. The present study paves the way for subsequent investigations into the molecular mechanisms regarding cellular interactions and responses, as well as the correlation between virulence factors and the induction of mucus production/expression during aEPEC infections.

## 1 Introduction

*Escherichia coli* is a bacterial species that is part of the microbiota of healthy individuals. However, some *E. coli* strains can also cause diseases in several (intra or extraintestinal) host sites (Nataro and Kaper, 1998; Andreas et al., 2013; Croxen et al., 2013; Sanchez-Villamil and Navarro-Garcia, 2015; Gomes et al., 2016; Martinson and Walk, 2020). Diarrheal diseases can be caused by *E. coli* and are currently among the ten leading causes of death worldwide, affecting mainly low-income countries (World Health Organization, 2020). *E. coli* strains associated with intestinal infections are known as diarrheagenic *E. coli* (DEC) (Nataro and Kaper, 1998; Gomes et al., 2016). According to genotypic and phenotypic characteristics associated with different combinations of virulence genes and the symptoms they generate in the host, DEC strains are classified into different pathotypes (Kaper et al., 2004; Croxen et al., 2013).

Enteropathogenic *E. coli* (EPEC), one of the DEC pathotypes, is characterized by the formation of attaching and effacing (A/E) lesions, in which the intimate adherence of the bacteria, mediated by the adhesive protein intimin, promotes a modification in the conformation of the enterocytes’ cytoskeleton. This modification is characterized by the accumulation of long actin filaments underneath the adhesion region and effacement of the cell’s microvilli, leading to the appearance of pedestal-like structures (Moon et al., 1983; Kaper et al., 2004; Sanchez-Villamil and Navarro-Garcia, 2015). The genes that are involved in the establishment of the A/E lesion are encoded in a pathogenicity island (PAI) called the locus of enterocyte effacement (LEE) (McDaniel et al., 1995; McDaniel and Kaper, 1997).

EPEC strains are subdivided into typical (tEPEC) and atypical (aEPEC) according to the presence or absence of a virulence-associated plasmid called pEAF that contains the EPEC adherence factor (EAF) sequence (Kaper et al., 2004). This plasmid encodes the type IV fimbria called the bundle-forming pilus (BFP), which is present in tEPEC, while aEPEC strains do not have this or have a defective fimbria operon and consequently promote different adherence phenotypes from the tEPEC (Trabulsi et al., 2002; Hernandes et al., 2009; Gomes et al., 2016).

tEPEC strains cause acute diarrheal episodes in children younger than two years in low-middle-income countries (Trabulsi et al., 2002). In contrast, aEPEC strains are isolated from individuals of all age groups, and infections are seen in developed and developing countries (Bhatt, 2021). aEPEC is a significant cause of diarrhea in children under five years in developing countries (Estrada-Garcia et al., 2009; Hernandes et al., 2009; Hazen et al., 2013; Dias et al., 2016; Hazen et al., 2016; Vieira et al., 2016; Ori et al., 2019). In addition, infected individuals may exhibit various clinical symptoms ranging from acute to persistent diarrhea (Hernandes et al., 2009; Hu and Torres, 2015). Comparative genomic analyses showed that the aEPEC genome is permissive (Afset et al., 2008; Bando et al., 2009), leading to the pathotype’s great heterogenicity. In some circumstances, this heterogenicity can be related to diverse host symptoms or phenotypes identified *in vitro* or *in vivo* (Hernandes et al., 2020).

To protect the mucosa, the host produces a thick, complex layer of mucus that covers the gastrointestinal tract in the stomach, small intestine, and large intestine (Birchenough et al., 2015). Mucus is composed of glycosylated proteins, mucins, enzymes, and antimicrobial peptides, but depending on its location in the gastrointestinal tract, it can have different compositions and conformations (Ermund et al., 2013; Birchenough et al., 2015). Although mucin secretion is constitutive, certain circumstances can lead to changes in its expression. During inflammatory diseases (as in Inflammatory Bowel Diseases (IBD) (Pereira et al., 2024) or cancerous processes, changes in mucin expression and/or secretion can generate pathophysiological conditions, such as diarrhea (Andrianifahanana et al., 2006).

In a previous study, our group demonstrated that two aEPEC strains (0421-1 and 3991-1) had a unique ability to significantly increase mucus production and secretion in the ligated rabbit ileal loop model *in vivo* (Vieira et al., 2010). This phenomenon contrasted with the interaction observed in the tEPEC prototype strain E2348/69, which did not induce this phenomenon (Vieira et al., 2010).

Different studies have reported some factors produced by Gram-negative and Gram-positive bacteria that induced mucus production in the host (Moal et al., 2002). Pic (a protein involved in intestinal colonization), secreted by pathogens of the Enterobacteriaceae family, is an important example of a mucus secretagogue (Navarro-Garcia et al., 2010). Furthermore, several species of microorganisms can regulate the increase in mucus production by inducing the expression of mucin genes (Sheikh et al., 2022). *Pseudomonas aeruginosa* and *E. coli* increase MUC2, MUC5AC, and MUC5B by activating the NF-κB pathway; some species from the microbiota (*Bifidobacterium dentium*, *Ruminococcus gnavus*, and *Lactobacillus rhamnosus*) regulate both the production of mucins and mucosal defense factors produced by goblet cells (Bergstrom et al., 2008).

The production of mucus is a natural defense mechanism that helps keep bacteria away from the cell’s surface and contributes to eliminating pathogens in mucosal areas of the body. However, some bacterial species have developed the ability to break down mucus, known as mucinolytic capacity (Navarro-Garcia et al., 2010; Hews et al., 2017). The degradation of the barrier formed by intestinal mucus is an essential process for bacterial pathogenesis. This process enables direct contact between bacteria and intestinal epithelial cells, which can subsequently lead to bloodstream permeation (Kim and Ho, 2010; Sperandio et al., 2013). In addition, these activities release less complex carbohydrates that can be used for bacterial nutrition (Flores-Sanchez et al., 2020).

This study aimed to investigate the ability of aEPEC strains to alter mucus production and determine if they have mucinolytic activity. For this purpose, we sequenced and compared the genomes of aEPEC strains that induce mucus production with strains that lack this property.

## 2 Materials and methods

### 2.1 Bacterial strains

Thirteen strains of aEPEC were included in this study (Vieira et al., 2001; Santos et al., 2019; Romão et al., 2024). **Table 1** presents their original serotype as detected by standard agglutination methods with somatic (O) and flagellar (H) specific antisera O1 to O173 and H1 to H52, provided by the Centers for Disease Control and Prevention (C.D.C., Atlanta).

**Table 1:** aEPEC strains used in this study.

Strain	Serotype expressed*	Strains reference	Genome Reference
51	O119:H2	Vieira et al., 2010	This study
70	O128ac:H2	Vieira et al., 2010	This study
0421-1	O101:H33	Vieira et al., 2001	This study
1582-4	O162:H-	Vieira et al., 2001	This study
1711-4	O51:H40	Vieira et al., 2001	Romão et al., 2024
2012-1	O26:[H11]	Gomes et al., 2004	Santos et al., 2019
2531-13	ONT:H-	Gomes et al., 2004	Santos et al., 2019
2731-1	ONT:H-	Vieira et al., 2001	This study
3391-3	ONT:H40	Vieira et al., 2001	This study
3881-3	O63:H6	Gomes et al., 2004	Santos et al., 2019
3991-1	ONT:H-	Gomes et al., 2004	This study
4361-1	O51:H40	Vieira et al., 2001	This study
4581-2	ONT:H40	Gomes et al., 2004	Santos et al., 2019

*Original identification, based on serological typing with O and H antisera provided by the Centers for Disease Control and Prevention (C.D.C.), Atlanta, Ga., U.S.A.

The aEPEC strains were isolated from fecal samples of children and collected during epidemiological studies on diarrhea conducted by our laboratory at the Federal University of São Paulo (UNIFESP), Brazil. They are stored at -80°C in Lysogeny broth (LB) containing 15% glycerol.

Among the 13 studied strains, two (0421-1 and 3991-1) induced mucus production in the rabbit ileal loop model, and five did not (1711-4, 3391-3, 3881-3, 4361-1, and 4581-2) (Vieira et al., 2010). This phenotype was unknown for the remaining six strains, which were chosen randomly.

### 2.2 Genome sequencing and genetic analysis

#### 2.2.1 Genome sequencing

Eight aEPEC strains were sequenced for the present study by the MicrobesNG sequencing service (Birmingham Research Park, Birmingham, UK - https://microbesng.com), which performed the extraction, library preparation, and genome sequencing and assembly. DNA was extracted and quantified using the Quant-iT dsDNA HS assay kit and the AF2200 plate reader (Eppendorf, Germany). Genomic libraries were prepared using Nex tera XT Library Prep Kit (Illumina, San Diego, U.S.A.), with 2 ng DNA and 1 min and 30 sec-elongation. Libraries were sequenced using the Illumina platform to obtain 2 x 250 bp paired reads. Genome sequencing achieved a minimum coverage of 80 x. Genomes were assembled by the *de novo* method using the SPAdes software (version 3.7) (Bankevich et al., 2012). All genomes, including the previously published ones, were annotated using Prokka (version 1.11) (Seemann, 2014).

#### 2.2.2 General genomic analyses

Genomic analyses were performed at the Center for Genomic Epidemiology (CGE) using services for the identification of acquired virulence genes (VirulenceFinder version 2.0) (Camacho et al., 2009; Joensen et al., 2014; Tetzschner et al., 2020), serotype (SerotypeFinder version 2.0) (Camacho et al., 2009; Joensen et al., 2015), and sequence type determination (M.L.S.T. version 2.0) (Wirth et al., 2006; Camacho et al., 2009) following the Warwick scheme. Virulence genes were also evaluated in the VFanalyzer (Liu et al., 2019). Identification of the phylogenetic group was performed using ClermonTyping (Beghain et al., 2018).

Three phylogenetic trees were built using Condon-tree methods performed by the Phylogenetic Tree Building Service at BV-BRC (Bacterial and Viral Bioinformatics Resource Center) (Wattam et al., 2017) in which the amino acid and nucleotide sequences of 1,000 single copy CDS (coding sequences) were concatenated and analyzed using RAxML (maximum likelihood randomized accelerated matrix). One three was built using 89 *E. coli* strains (**Supplementary Table 1**), well documented and previously published, belonging to EPEC global lineages (Hazen et al., 2013; Ingle et al., 2016; Hernandes et al., 2020) and the 13 aEPEC strains from the present study to determine to which of the global EPEC/EHEC lineage the strains belong. The *E. fergusonii* ATCC 35469 was used as an outgroup for the global lineages tree.

The second phylogenetic tree was constructed using public genomes deposited at the NCBI that showed high genomic similarity with strains 0421-1, 1582-4, 2531-13, and 3991-1, identified in BV-BRC by the Similar Genome Finder service that uses the MASH/MinHash algorithm, as previously published (Santos et al., 2021). Fifty genomes were searched for each strain using a *p*-value < 0.001 and a maximum distance of 0.01 as search parameters against all public *E. coli* genomes available in the database as of January 2022. The identified genomes were pooled, and 64 unique genomes (**Supplementary Table 2**) were evaluated together with the four aEPEC strains which belong to ST378 (0421-1, 1582-4, 2531-13, and 3991-1). For this phylogenetic tree, the *E. fergusonii* ATCC 35469 and four DEC prototype strains (ETEC str H10407 – ST10; O104:H4 str. 2011C-3493 – ST678; O127:H6 str. E2348/69 – ST15; and O157:H7 str. Sakai ST11) were used as outgroups.

The third phylogenetic tree was built using only the 13 aEPEC strains evaluated in the present study and it was supplemented with virulence factors retrieved from VFanalyzer tool from VFDB, which were used to create a heatmap to compare known virulence genes present in the strains. The final layout of the phylogenetic trees was constructed using iTOL (version 6) (Letunic and Bork, 2021).

The NCBI’s BLASTP (Altschul et al., 1997) was used for intimin typing and searching for previously described bacterial mucinases (**Table 2**). Finally, pangenome analyses of nine aEPEC strains (0421-1, 1582-4, 1711-4, 2531-13, 3391-3, 3881-3, 3991-1, 4361-1, and 4581-2) were performed using Roary (Page et al., 2015).

**Table 2:** Previously described bacterial mucinases investigated in the aEPEC strains of this study.

Protein (species)	Accession number	Protein length (aa)
Autotransporter protein Sha (*Escherichia coli*)	QDB64255.1	1302
Autotransporter protein TSH (*E. coli*)	Q47692.1	1377
Autotransporter protein Vat (*E. coli* AIEC)	CAP74833.1	1376
Autotransporter protein Pic (*E. coli* EAEC)	Q7BS42.2	1372
Lipoprotein metalloprotease SslE (*E. coli* – DEC variant)	WP_105458559.1	1519
Lipoprotein metalloprotease SslE (*E. coli* – UPEC variant)	WP_001034562.1	1520
Metallopeptidase StcE (*E. coli* EHEC)	WP_001358886.1	898
Hemagglutinin/proteinase HapA (*Vibrio cholerae*)	WP_142740896.1	609
Lipoprotein metalloprotease Acf (*Vibrio mimicus*)	WP_000865235.1	1526
Peptidase M60, enhancin and enhancin-like (*Streptomyces* sp.)	SCX85042.1	463
Metallopeptidase M60 (*Bacillus wiedmannii*)	WP_064057603.1	1064
M60-like BT4244 (*Bacteroides thetaiotaomicron*)	WP_008764444.1	857
ImpA family metalloprotease (*Pseudomonas aeruginosa*)	WP_034013903.1	923
Peptidase M60 domain-containing protein (*Akkermansia muciniphila*) – Chain A	7YX8_A	441
Peptidase M60 domain-containing protein (*A. muciniphila*) – Chain B	7YX8_B	441
Amuc_0908 (*A. muciniphila*)	ACD04740.1	747
Amuc_1514 (*A. muciniphila*)	ACD05335.1	660
Immuno-dominant variable surface antigen (*Entamoeba histolytica*)	XP_654508.1	1113
Immuno-dominant variable surface antigen-like (*Trichomonas vaginalis*)	XP_001330197.1	1148
ZmpB metallopeptidase (*Clostridium perfringens*)	5KDN_A	530

### 2.3 Rabbit-ligated ileal loop model

To test the ability of aEPEC strains to induce increased mucus production *in vivo*, we used the rabbit-ligated ileal loop assay, as described previously (Vieira et al., 2010). Before surgery, a male New Zealand white rabbit (7 weeks old, weighing 1.8 kg) was screened for the absence of coccidia by parasitological examination. The absence of the *eae* gene, which encodes intimin, was confirmed by PCR because rabbits can be infected by specific EPEC strains (REPEC, rabbit EPEC). Briefly, after 24 h of fasting, the rabbit was anesthetized with a combination of acepromazine (0.5 mg/kg) (0.2% Acepran^®^ Uni-vet S/A, São Paulo – SP, Brazil) and Ketamine (5 mg/kg) intramuscularly, followed by deep inhalation anesthesia with isoflurane (1.5%) throughout the procedure. After ventral laparotomy, the distal portion of the ileum (middle ileum) was washed thrice with 20 mL of sterile saline. Segments of the ileum, measuring approximately 5 cm in length each, were ligated at both ends with a 4.0 cotton suture, with approximately 3 cm between the loops. An inoculum of 0.3 mL of bacterial suspension (~1×10^8^ CFU/mL) or sterile LB medium was inoculated into each loop, with one loop per strain (aEPEC 1582-4, 2531-13, and 3991-1) or sterile LB. After the loops were inoculated, the animal’s abdomen was sutured. Morphine (2.5 mg/kg subcutaneously or intramuscularly) was administered every 4 hours for postsurgical analgesia. Saline fluid therapy was administered subcutaneously (20 mL, once). After 18 hours, the rabbit was euthanized with an overdose of anesthesia (6% isoflurane and 20 mg/kg potassium chloride) (Ministry of Science, Technology, and Innovation, 2018).

After 18 hours of infection and subsequent euthanasia, a ventral laparotomy was performed again to remove the ligated ileum containing the inoculated loops and subsequently transferred to a container with sterile saline. The loops were handled in a laminar flow using sterile surgical scissors and forceps to remove fat tissue. The cleaned ileum was placed on a contrasting-colored surface (blue) to facilitate photographic documentation of mucus accumulation. After the photographic documentation, a small incision was made near the ligature to assess the internal content of each loop, and a new photo documentation was performed. Those strains that promoted loop distention and mucus production compared to the negative control were considered positive. The assay was performed using three animals, and each strain was evaluated once per animal. Therefore, the assay was performed in three biological replicates.

The animal used for experimentation was handled following the current standards by Dr. Terezinha Knöbl from the Veterinary Faculty of the University of São Paulo. The Federal University of São Paulo Ethics Committee approved the animal experiment (n° 5161030820).

### 2.4 Assessment of the mucinolytic activity of aEPEC strains

To evaluate the mucinolytic ability of aEPEC strains, 13 strains underwent *in vitro* degradation studies using two approaches: mucin consumption as the sole substrate and bacterial penetration across a mucin layer.

The mucin consumption test protocol was adapted from a study by Sauvaitre et al. (2022) with some modifications. Overnight bacterial cultures were diluted 1:10 in two fresh LB-containing tubes and grown for about an hour at 37°C with 120 rpm shaking to reach a mid-log phase (OD_600_ 0.45), which is equivalent to 1 × 10^8^ CFU/mL. The bacteria were then pelleted at 3500 rpm for 10 minutes, washed with 1x PBS, and resuspended in an equal volume of M9 minimal medium (M9) with and without 3 g/L of commercial mucin-type III (Sigma-Aldrich, St. Louis, MO, U.S.A.). The mixture was then incubated at 37°C with shaking for 6 hours.

An aliquot of both control and treated media was taken at 0, 2, 4, and 6 hours of incubation, serially diluted in 1x PBS, plated onto LB agar, and incubated overnight at 37°C. It is important to note that only salts were included in M9 media without carbon sources.

The Transwell^®^ membrane system was used to evaluate bacterial mucus penetration, as previously reported (Flores-Sanchez et al., 2020), with modifications. The Thincert™ chambers with PET microporous membrane of 8-µm (Greiner Bio-One, Frickenhausen, Germany) were prepared with 50 µL of commercial mucin (100 g/L) and 200 µL of Dulbecco Modified Eagle Medium (DMEM) High Glucose (Gibco, Grand Island, NY, U.S.A.) forming a thick layer of approximately 2.5 mm, and placed into 24-well cell culture plates (Corning Inc., Kennebunk, U.S.A.) containing 1 mL of LB. The bacterial suspension used for the inoculum test was 1 × 10^7^ CFU, similar to the suspension used in the consumption test. The system was incubated statically at 37°C for 3 hours. Afterward, a sample was taken from each lower compartment, diluted, plated, and left to grow overnight at 37°C.

For both assays, positive controls, including EAEC 042 and tEPEC E2348/69, and the negative controls, *E. coli* K-12 strains MA3456 and DH5α, were used. Additionally, each treatment consisted of three independent experiments in triplicate.

The protocol described by Sauvaitre et al. (2022) with modifications was used. Briefly, agar layers containing 400 µl of 1% bacteriological agar with and without commercial mucin (50 g/L) in 24-well cell culture plates were used to determine the specificity of aEPEC adhesion to mucin. To perform that, a bacterial inoculum of 1 × 10^7^ CFU of each sample diluted in 1×PBS was applied into both wells (agar alone and agar mucin) and incubated for 1 h at 37°C with 100 rpm shaking. After three washes with 1× PBS, the agar layers were carefully collected and placed into 15 mL tubes containing 10 mL of 1× PBS and mixed using a vortex for 30 seconds. The bacterial counting protocol was the same as explained in the previous test. Two independent experiments with technical replicates were accomplished using the ETEC H10407 strain as a positive control.

## 3 Results

### 3.1 Genomic investigation of aEPEC strains

**Figure 1:**
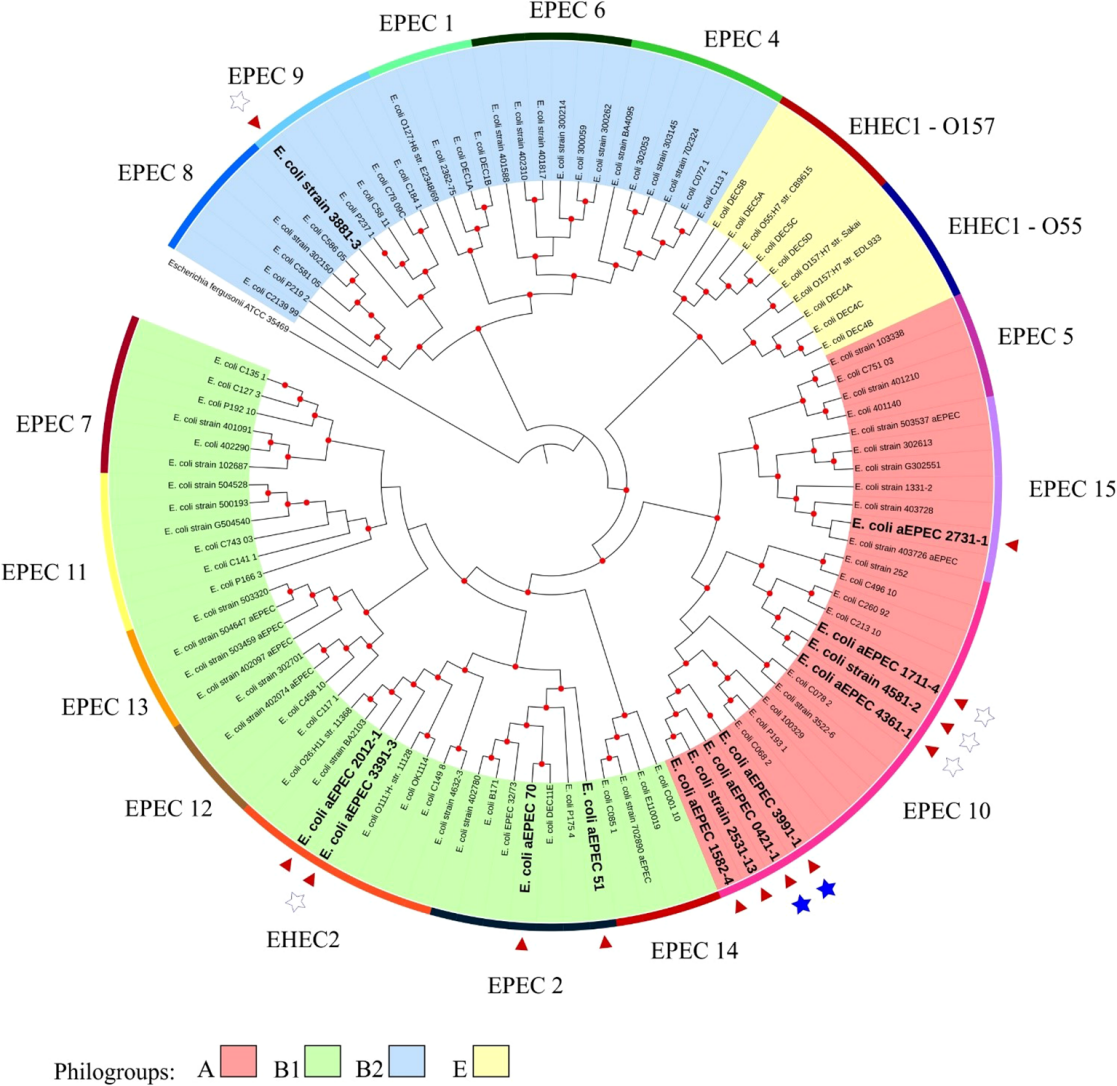
Phylogenetic tree containing the EPEC and EHEC genomes representing the global clonal groups. The tree was constructed using the codon tree methodology by evaluating 1,000 single-copy coding genes in a RA×ML matrix. The colors of the clades represent the phylogenetic origin, and the colors of the strips are the clonal (EPEC) groups. The 13 aEPEC strains from this study are highlighted by a red arrow and bold large letters. Stars indicate the strains tested in the rabbit ileal loop model: the filled stars represent strains that induced mucus secretion, and empty stars represent the non-mucus inducer strains. The *Escherichia fergusonii* ATCC 35469 strain was used as the root for structuring the tree. Red dots at tree nodes indicate bootstrap ≥80.

The minimum mean coverage of 89 × was obtained in the genome sequencing of eight aEPEC strains in the present study (**Supplementary Table 3**). The genome predicted sizes of the 13 aEPEC strains studied ranged from 4,707,164 to 5,659,834 bp (**Table 3**). The genomic analysis of aEPEC strains revealed that they belonged to phylogroups A, B1, and B2 and had five types of intimin (α, β, ι2, ρ, and θ). These strains were distributed in seven different STs, as shown in **Table 4**. Among these strains, 0421-1 and 3991-1 were known to induce mucus production in the rabbit ileal loop. These two strains shared some genomic features, including phylogroup A, ST378, genomic serotype O101/O162:H33, and intimin ι2 subtype. Interestingly, two other strains, 1582-4 and 2531-13, also shared these features, indicating they may induce mucus production (**Table 4**). To determine the evolutionary relationships among the studied aEPEC strains, a phylogenetic tree was built using the genomes published in three studies, which characterized the most prevalent EPEC and EHEC global lineages worldwide and the Brazilian aEPEC strains (Hazen et al., 2013; Ingle et al., 2016; Hernandes et al., 2020) (**Figure 1**).

**Table 3:** General genomic characteristics of the 13 aEPEC strains studied.

Strain	Genome size (bp)	CG (%)	CDS	L50	N50	Contigs	Genbank Accessions	Genome Reference
51	5202039	50.44	5559	13	117413	223	GCA_036927655.1	Present study
70	4985719	50.65	5169	13	126825	141	GCA_036927775.1	Present study
0421-1	5105331	50.68	5584	18	100698	327	GCA_036927625.1	Present study
1582-4	5110639	50.61	5701	19	99133	434	GCA_036925215.1	Present study
1711-4	4722189	50.45	5435	1	4696149	4	GCA_031460355.1	Romão et al., 2024
2012-1	5616016	50.42	6058	20	97093	260	GCA_003651405.1	Santos et al., 2019
2531-13	5092005	50.58	5465	21	72340	376	GCA_003651425.1	Santos et al., 2019
2731-1	5042367	50.50	5363	16	121400	227	GCA_036925195.1	Present study
3391-3	5659834	50.46	6449	16	131552	474	GCA_036927645.1	Present study
3881-3	4718198	50.72	4717	8	241425	115	GCA_003651355.1	Santos et al., 2019
3991-1	4801192	50.84	5270	19	76240	380	GCA_036927635.1	Present study
4361-1	4706115	50.42	4813	8	210156	60	GCA_036927615.1	Present study
4581-2	4707164	50.41	4702	10	136790	105	GCA_003651325.1	Santos et al., 2019

**Table 4:** Genomic classification of the aEPEC strains studied.

Phylogroup	Intimin subtype	Sequence Type	Serotype^a^	Original Serotype^b^	Strain
**A**	θ (theta)	10	O51:H40	O51:H40	1711-4
				O51:H40	4361-1
				ONT:H40	4581-2
	ρ (rho)	301	O180:H2	ONT:H-	2731-1
	**ι 2 (iota 2)**	**378**	**O101/O162:H33**	**O101:H33**	**0421-1**
				**O162:H-**	**1582-4**
				**ONT:H-**	**2531-13**
				**ONT:H-**	**3991-1**
**B1**	β (beta)	3	O128ac:H2	O128ac:H2	70
		20	O119:H2	O119:H2	51
		29	O26:H11	O26:H-	2012-1
			O123/O186:H11	ONT:H40	3391-3
**B2**	α (alpha)	583	O63:H6	O63:H6	3881-3

a new serotype determined based on genome analysis.

b original identification, based on serological typing with O and H antisera provided by the Centers for Disease Control and Prevention (C.D.C.), Atlanta, Ga., U.S.A.

Bold data highlight shared features from the mucus inducing strains (0421-1, 3991-1) with the other two aEPEC strains identified (1582-4 and 2531-13).

**Figure 2:**
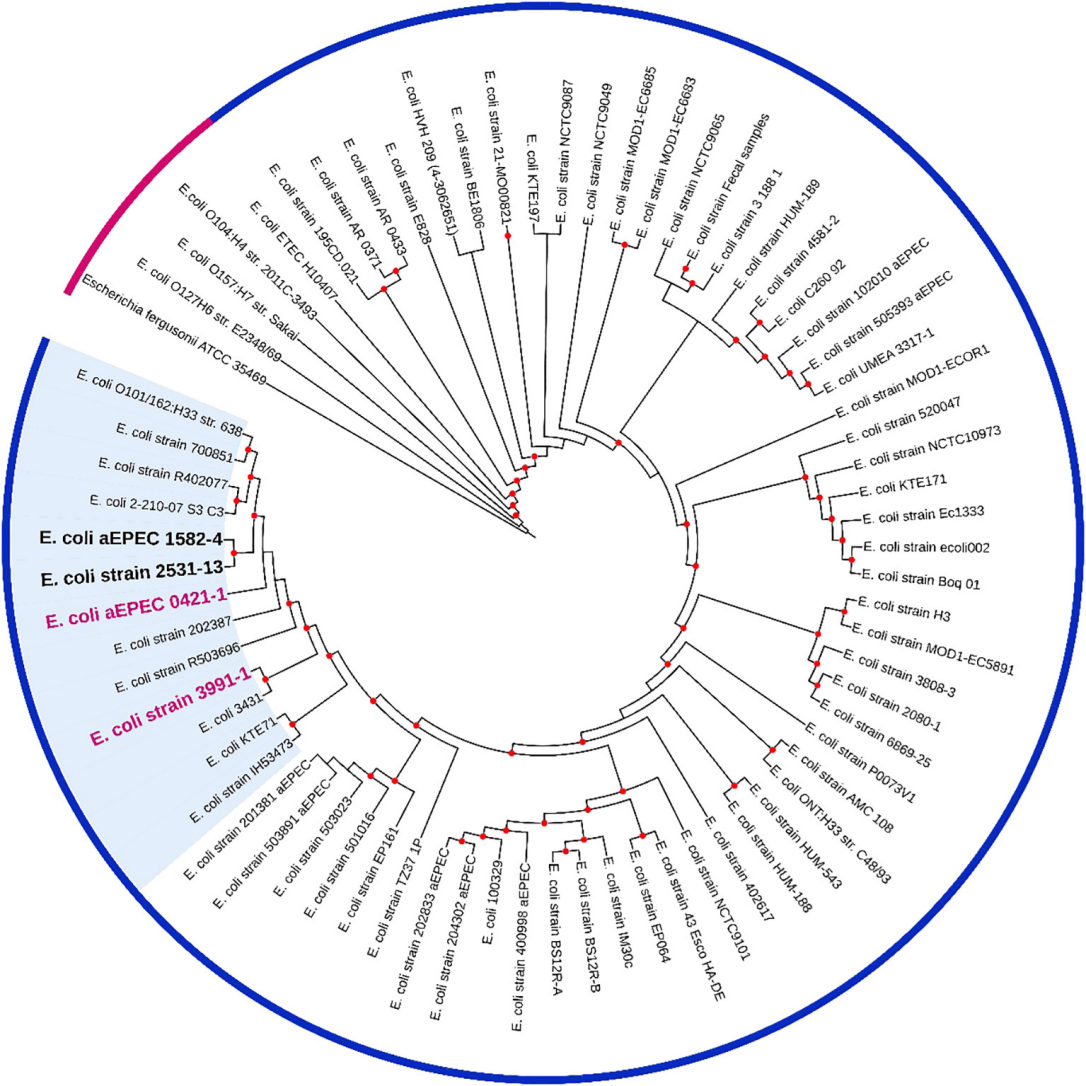
A phylogenetic tree containing 64 *E. coli* genomes publicly available and similar to mucus inducers aEPEC strains from ST378 (0421-1, 1582-4, 2531-13, and 3991-1). The tree was built using the codon-tree method evaluating 1,000 CDS in an RA×ML matrix. Bootstraps ≥80 are shown as red dots. Pink strip, prototype strain from the outgroup; blue strip, indicates all strains retrieved by MASH and belonging to the clonal complex MLST10 (CC10). A bold pink label was used for strains 0421-1 and 3991-1, and a bold black label for strains 1582-4 and 2531-13. The cluster of strains highly similar to 0421-1 and 3991-1 are on a blue background. The metadata of the strains are in **Supplementary Table 2**.

Based on this analysis, it was possible to verify that the aEPEC strains of the present study were distributed in five clonal groups: EPEC 2 (51 and 70), EPEC 9 (3881-3), EPEC 10 (0421-1, 1582-4, 1711-4, 2531-13, 3991-1, 4361-1, and 4581-2), EPEC 15 (2731-1), and EHEC 2 (2012-1 and 3391-3). It is worth noting that strains 0421-1, 1582-4, 2531-13, and 3991-1 belong to the same global group (EPEC10) and are clustered together, which suggests a clonal relationship among them (**Figure 1**). To verify how closely related these four strains were, a phylogenetic tree enriched with genomes similar to each of these strains was constructed to evaluate this close relationship. Mash was used to identify 50 similar genomes to each of the 4 aEPEC strains. The genomes were pooled, and 64 unique genomes identified were used together with aEPEC 0421-1, 1582-4, 2531-13, and 3991-1 to build a specific tree that confirmed the clonal proximity of the strains (**Figure 2**). The four strains clustered in one branch with the other nine aEPEC strains. While strains 1582-4 and 2531-13 were grouped closer to 0421-1, the aEPEC 3991-1 was more distant from them on the branch’s base (**Figure 2**).

### 3.2 Analysis of mucus production *in vivo*

**Figure 3:**
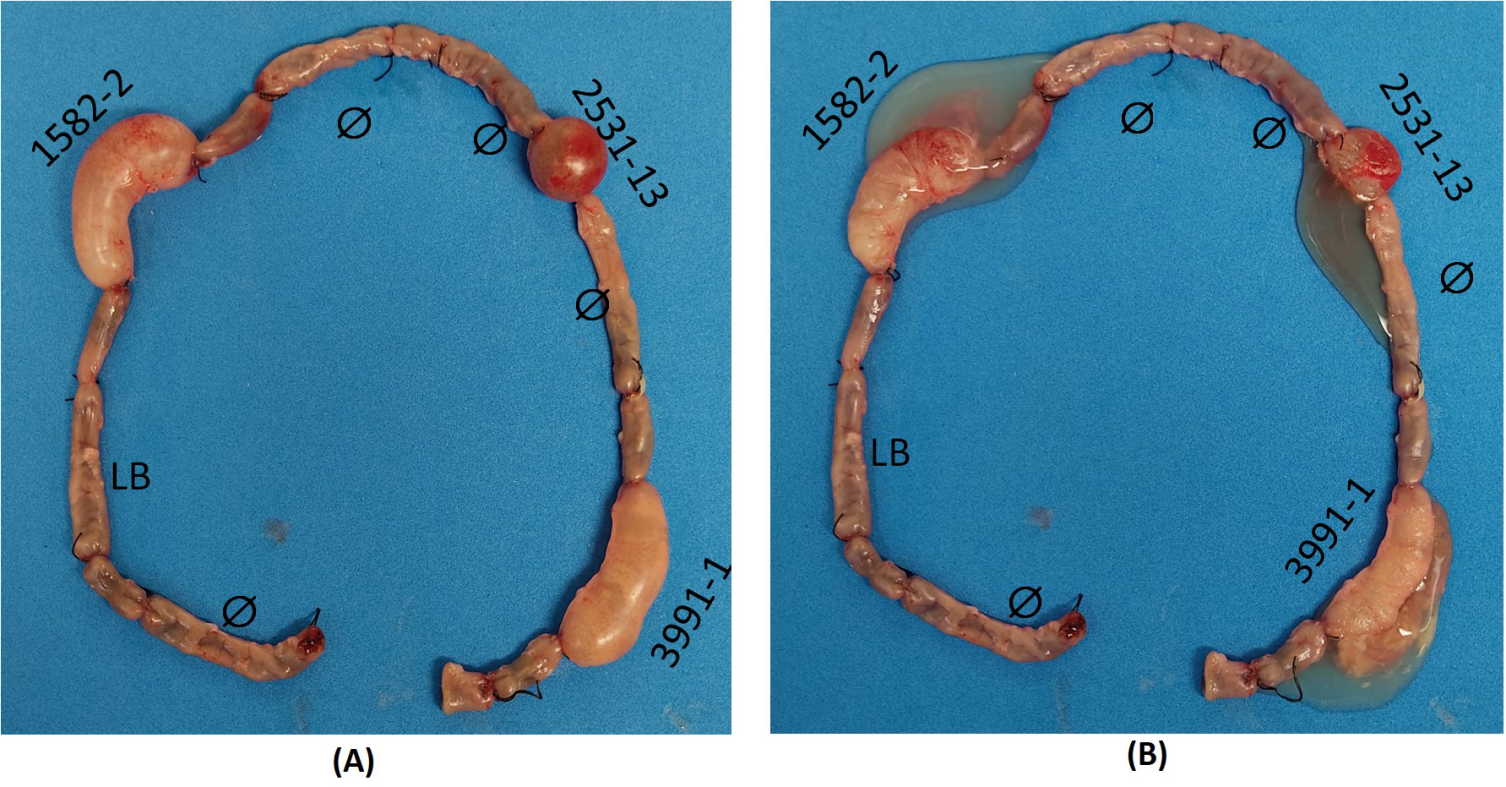
Rabbit ileum with ligated loops after 18 h of inoculation *in vivo*. Clockwise: initial empty loop (Ø); intermediate loop (uninoculated); loop inoculated with sterile Lysogeny broth (LB) (negative control); intermediate loop; loop inoculated with aEPEC 1582-4 strain; intermediate loop; empty loop (Ø); intermediate loop; empty loop Ø; loop inoculated with aEPEC 2531-13 strain; empty loop Ø; intermediate loop; loop inoculated with aEPEC 3991-1 strain (positive control); final empty loop. **(A)** Original loops: only aEPEC infected loops are swollen **(B)** Open loops showing the mucous fluid content of each infected loop.

The rabbit ligated ileal loop model was performed with the aEPEC strains 1582-4 and 2531-13 to investigate whether they also induced mucus production since they belonged to the same cluster of the mucus-inducer strains 3991-1 and 0421-1. After 18 hours of infection and removal of the rabbit’s ileum from the peritoneal cavity, it was observed that the strains 1582-4 and 2531-13, and the positive control strain 3991-1, induced a significant increase in mucous in the loops where they were inoculated. In contrast, the inoculation of sterile LB did not cause mucus production, and there was no such increase in the non-inoculated loops, as seen in **Figure 3**.

### 3.3 Characterization of aEPEC strains regarding virulence factors

Considering the vast genomic diversity of the aEPEC strains, we carried out comprehensive research on the virulence factors (VFs) present in the strains to identify their similarities and differences. We highlighted only genes with complete operons.

**Figure 4:**
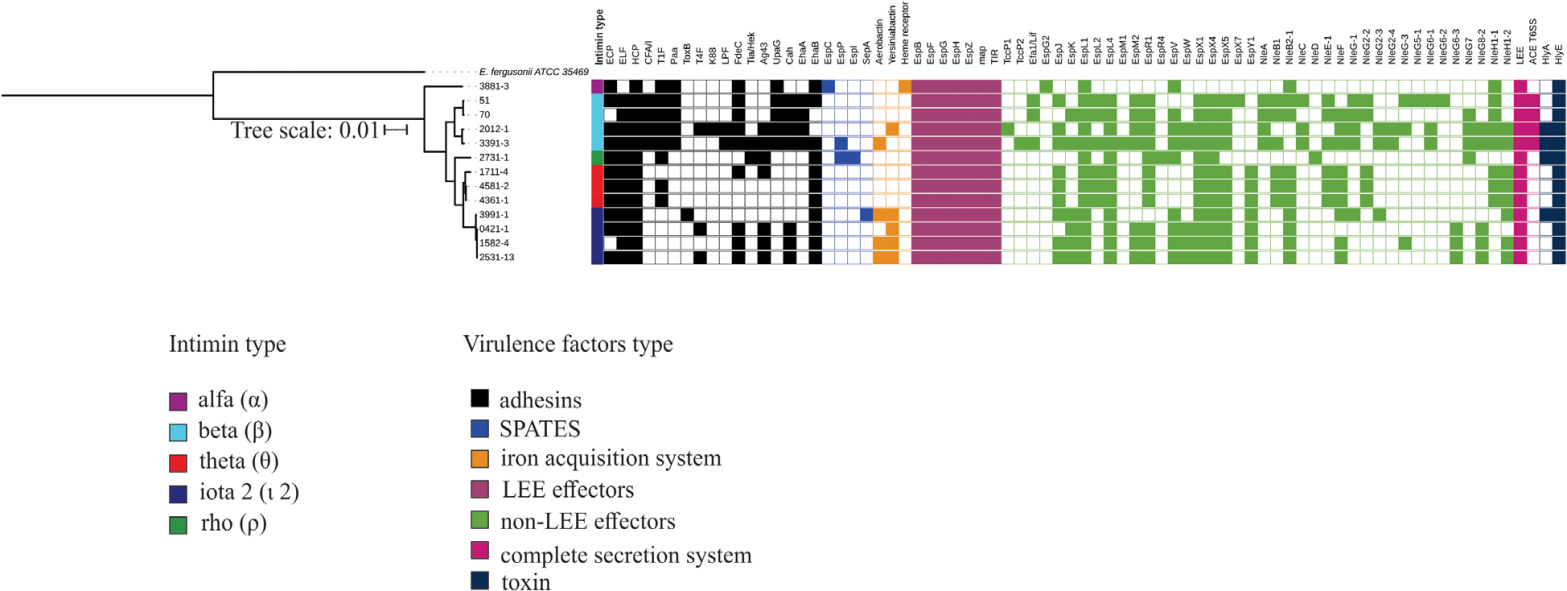
Complete virulence factor-encoding operons and sequences identified in the genome of the 13 aEPEC strains evaluated. The phylogenetic tree was built using these strains and added to the VF profile, which was determined using the VFanalyzer search tool from VFDB. The colored strip represents the type of intimin present in the strains. Only VFs that occurred at least in one strain were displayed in the tree.

Some VFs were present in all 13 strains, such as the HCP and intimin adhesins, cytolysin A toxin, the type three secretion system (T3SS)-encoding genes in the LEE region, the LEE effectors, EspB, EspF, EspG, EspH, EspZ, Map, and Tir, and the non-LEE effector EspL1. However, no autotransporter protein or any specific type of nutrient uptake system was shared among all 13 aEPEC strains that were analyzed (**Figure 4**).

Despite the genetic identity of the four mucus-inducing strains 0421-1, 1582-4, 2531-13, and 3991-1, not all the VFs were shared by all four strains (**Figure 4**). These strains shared a complete LEE region (EspB, EspF, EspG, EspH, EspZ, Map, Tir), and the adhesins ELF, HCP, and EhaB, Yersiniabactin, the non-LEE effectors EspK, EspL1, EspL4, EspV, EspX1, EspX4, EspX5, EspY1, and NleB2-1, and the hemolysin HlyE. However, none of the VFs searched were found exclusively in the mucus-inducing strains. A notable finding is that none of these strains possessed a complete type 1 fimbria operon. (**Figure 4**).

### 3.4 Comparative pangenome analyses

Considering that an extensive genomic analysis could help identify genes present in the mucus-inducing strains and absent in the non-inducing strains, a pangenome analysis was conducted in Roary to compare the genomes of the mucus-inducing strains (0421-1, 1582-4, 2531-13, and 3991-1) with the genomes of strains previously confirmed in the same model as non-inducing (1711-4, 3391-3, 3881-3, 4361-1, and 4581-2).

**Figure 5:**
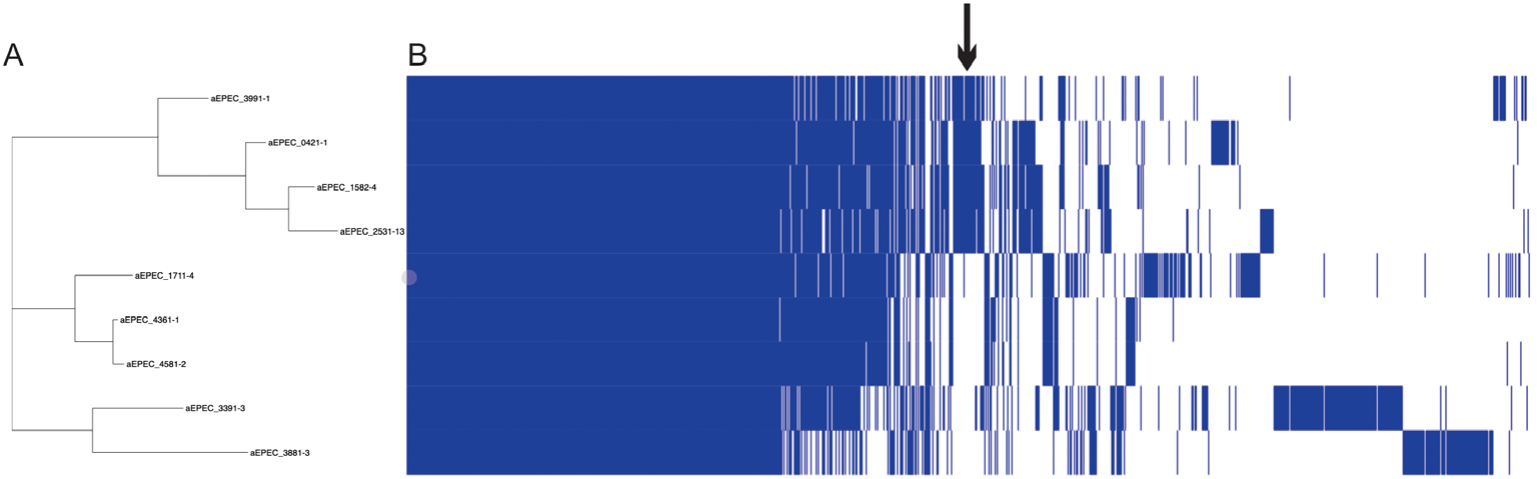
Pangenome analysis of 9 aEPEC strains. The figure displays **(A)** a SNP tree and **(B)** a corresponding matrix illustrating the presence and absence of core and accessory genes. The arrow indicates the region that concentrates the genes exclusive of mucus-inducing strains.

The pangenome of the nine aEPEC strains comprises 8819 genes, with a core genome of 2923 genes. Cloud and Shell genes comprise 3025 and 2871 genes, respectively. The phylogenomic tree retrieved from Roary grouped the mucus-inducing strains in one cluster. It showed that few genes are exclusively shared among strains that induce mucus production in the animal model (**Figure 5**; **Supplementary Table 4**). Evaluation of the genes in the presence and absence genes dataset of the two groups showed that 252 genes are shared and present exclusively in strains that induced mucus production in the ileal loop model. Most of the genes, 184, were annotated as hypothetical proteins, while the remaining 68 genes were variants of genes present in all strains, like the *eae* and *tir* genes that were different among the aEPEC strains studied (**Supplementary Table 4**), suggesting that genes related to mucus induction may be among the 252 genes identified exclusively in this group.

### 3.5 Screening for mucinases and SslE protein

Our research has led to a hypothesis that strains inducing increased mucus production may produce mucinases to degrade mucins. The search for known mucinases in the genome of all 13 aEPEC strains studied showed that four strains (51, 2012-1, 2731-1, and 3391-3) carry one putative protein with full coverage and high protein identity (varying from 95.9% to 96.4%) with the lipoprotein metalloprotease SslE (**Table 5**). The strain 70 showed a coverage of only 42%, suggesting that the protein is incomplete. The other eight strains, including the mucus-inducing strains, did not harbor any known mucinase in their genome.

**Table 5:** Comparison of the metalloproteinase SslE predicted protein of the aEPEC strains studied.

Strains	SslE (WP_105458559.1) ExPEC variant		SslE (WP_001034562.1) DEC variant	
	Protein cover (%)	Protein identity (%)	Protein cover (%)	Protein identity (%)
51	100	96.2	100	88.2
70	42	96.1	42	88.6
0421-1	–	–	–	–
1582-4	–	–	–	–
1711-4	–	–	–	–
2012-1	100	96.4	100	88.0
2531-13	–	–	–	–
2731-1	100	95.9	100	87.2
3391-3	100	96.4	100	88.0
3881-3	–	–	–	–
3991-1	–	–	–	–
4361-1	–	–	–	–
4581-2	–	–	–	–

### 3.6 Growth of aEPEC in M9 medium supplemented with mucin

**Figure 6:**
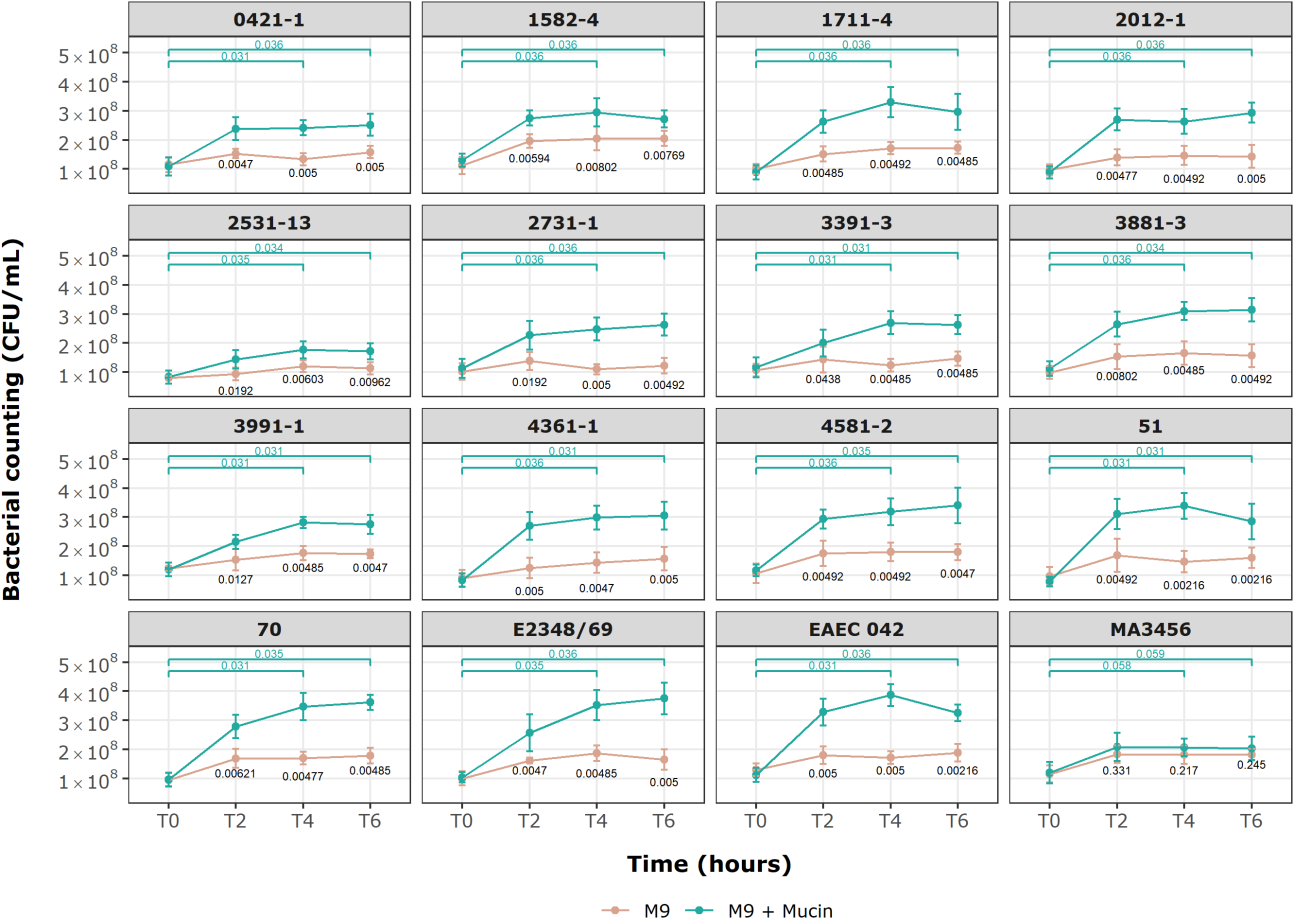
Growth kinetics of *E. coli* strains in minimal M9 medium with and without commercial mucin. Behavior of 13 aEPEC strains in M9 with mucin as sole carbon source. An initial inoculum of 1 x 10^8^ CFU/mL was evaluated over 6 h of incubation at specific time points. Data are expressed in mean ± S.D. and are representative of three independent experiments. Wilcoxon signed-rank test (green) showing p-values for paired groups (green), M9 and M9 + Mucin unpaired values (black p-values) by Mann-Whitney U test. Statistical analyses and visualization were conducted using ggplot2 in RStudio 2023.06.0.

Growth kinetic results in a medium supplemented with mucin for the selected aEPEC strains are provided in **Figure 6**. After a 6-hour incubation period, all *E. coli* strains could use mucin as a sole carbon source except the MA3456 negative control strain (p = 0.245, Mann-Whitney test). The initial viable cell count (1.05 x 10^8^ CFU/mL, mean) achieved values three to four times higher at 4 and 6 h of treatment for the following six aEPEC strains: 51, 70, 1711-4, 3881-3, 4361-1, 4581-2, as well as tEPEC E2348/69 and EAEC 042, which are the positive controls. None of the four mucus-inducing strains were included in this category. After 2 hours of incubation, only four strains (2531-13, 2731-1, 3391-3, and 3991-1) still had not achieved a significant bacterial growth rate relative to the M9 control condition (p > 0.01). However, all aEPEC strains showed a considerable growth rate after 6 h of incubation compared to the initial inoculum (p < 0.05, n = 9, Wilcoxon signed-rank test).

### 3.7 Bacterial penetration across a mucin layer

**Figure 7:**
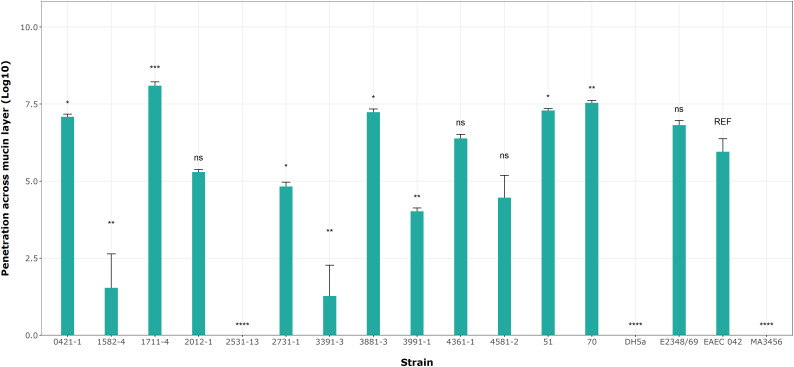
aEPEC strains’ ability to penetrate a mucin layer. Bacterial count expressed in log shows significant differences from the control strain EAEC 042. Data bars display means ± S.D. and represent three independent biological replicates. *p < 0.05, **p < 0.01, ***p < 0.001, one-way ANOVA with *post hoc* Tukey’s test analyzed in RStudio 2023.06.0. ns, not significant and **** p < 0.0001.

Based on the results of mucin degradation (Flores-Sanchez et al., 2020), we chose EAEC 042 as the reference strain for this assay. **Figure 7** shows significant differences in penetration levels by the aEPEC strains. Among the 17 strains analyzed (including controls), only three strains (1582-4, 2531-13, and 3391-3) were not able to penetrate the mucin layer (**Figure 7**; **Table 6**); the first one is a mucus-inducing strain. Five strains showed significantly higher levels of penetration compared to EAEC 042, with the 1711-4 strain having the best performance (p = 0.001), followed by the 70 (p = 0.01), 3881-3 (p = 0.02), 51 (p = 0.02), and 0421-1 (p = 0.029) strains; the latter one is a mucus-inducing strain. The results also showed no significant differences (ns) in the log values of three strains, 2012-1, 4361-1, and 4581-2 (ranging from 4.5 to 6.4), like that seen by the EAEC strain (5.95). In summary, we observed that 10 (51, 70, 0421-1, 1711-4, 2012-1, 2731-1, 3881-3, 3991-1, 4361-5, and 4581-2) of the 13 aEPEC strains crossed the dense mucin layer with log values ranging from 4.0 - 8.1, while the remaining strains (1582-4, 2531-13, and 3391-3) presented lower log counts ranging between 0 to 1.5. The prototype E2348/69 strain achieved a similar log value to EAEC 042 (ns). In contrast, the two *E. coli* K12 strains, MA3456 and DH5α, could not penetrate mucin.

**Table 6:** Summary of mucin hypersecretion, consumption, penetration, and adherence by aEPEC strains.

Strain Mucin	51	70	0421-1	1582-4	1711-4	2012-1	2531-13	2731-1	3391-3	3881-3	3991-1	4361-1	4581-2	E2348/69	MA3456
**Hypersecretion** **induction ^a^**	NT	NT	+	+	–	NT	+	NT	–	–	+	–	–	–	NT
**Consumption**	+	+	+	+	+	+	+	+	+	+	+	+	+	+	–
**Penetration ^b^**	+	+	+	–	+	+	–	+	–	+	+	+	+	+	–
**Adhesion ^c^**	–	+	–	–	+	–	–	+	–	–	–	–	+	–	–

^a^In the rabbit ligated ileal loop model: NT, not tested; ^b^in the Transwell test; ^c^adhesion in wells containing agar+mucin. +, positive; -, negative.

### 3.8 Adhesion assay of aEPEC strains on mucin agar plates

**Figure 8:**
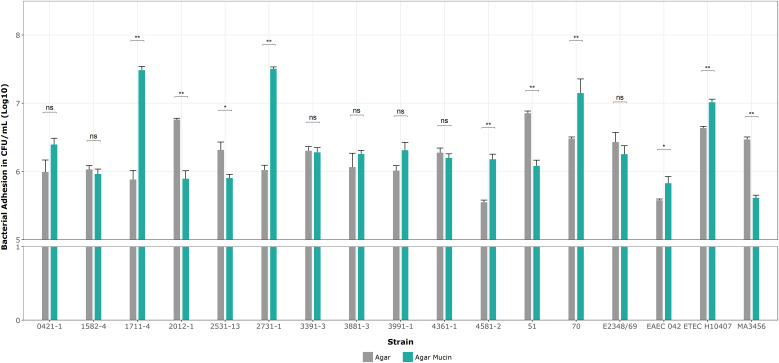
Binding assays on 1% agar plates with and without mucin. Data bars display means ± SD and represent two and three biological and technical replicates, respectively. *p < 0.05, **p < 0.01, ns as no significant, and Mann-Whitney U tests for unpaired groups were analyzed by RStudio 2023.06.0.

Next, we determined the specificity of the aEPEC strains’ adhesion to mucin on 5% mucin agar plates as complementary evidence for the penetration assays. **Figure 8** shows no significant differences in mucin binding in the mucus-inducing strains, except strain 2531-13, which showed more significant growth on agar without mucin. Only four strains showed significantly higher levels of adherence compared to their results on agar without mucin in the following order: 1711-4 (p = 0.002), 2731-1 (p = 0.004), 70 (p = 0.004), and 4581-2 (p = 0.005). Since the ETEC H10407 strain was also tested to validate our assay, their log means values of 7.01 versus 6.64 on agar alone (p = 0.004) were consistent with the reported by Sauvaitre et al. (2022).

**Table 6** summarizes all results related to the interaction of the 13 aEPEC strains regarding mucus hypersecretion *in vivo* (in the ligated rabbit ileum loop model) and mucin utilization. Considering the conditions tested, there was no direct correlation between mucus hypersecretion induction and mucin consumption, penetration, and adherence.

All aEPEC strains used mucins for their growth regardless of their ability to induce mucus hypersecretion. Additionally, two mucus-inducing strains (0421-1 and 3991-1) could penetrate the mucin barrier, while the other two (1582-4 and 2531-13) could not. In contrast, four of the five non-mucus-inducing strains (1711-4, 3391-3, 3881-3, 4361-1, and 4581-2) penetrated the mucus barrier efficiently.

## 4 Discussion

The secreted mucus layer is the first defense against pathogens that need to adhere to and colonize the gut and promote disease since it imposes a physical barrier that avoids direct bacterial contact with the gut cells (Moal and Servin, 2006; Sicard et al., 2017; Cai et al., 2020). However, bacteria from the microbiota can adhere to, penetrate, and break mucins for consumption, using the fluidic mucus layer as the preferred colonization niche (Sicard et al., 2017). On the other hand, pathogens can interact with the mucus layer in different ways, ranging from complete degradation to only attachment. Understanding these interactions is crucial for enhancing infection control and reducing the high mortality rates of diarrhea and other intestinal diseases worldwide.

The differences between typical EPEC (tEPEC) and atypical EPEC (aEPEC) strains in phenotype, molecular characteristics, epidemiology, and pathology are well established. aEPEC strains exhibit more significant phylogenetic heterogeneity and a broader diversity of VFs (Trabulsi et al., 2002; Hernandes et al., 2009; Hu and Torres, 2015; Bhatt, 2021; Santos et al., 2021).

In a previous study, it was shown that two aEPEC strains (0421-1 and 3991-1) induce mucus hyperproduction *in vivo* in the rabbit’s ligated ileal loop model and HT29-MTX cells *in vitro*, where the mucus secretion resulted from an increase in the expression of the mucin genes *MUC5AC* and *MUC4* (Vieira et al., 2010). The data suggested a conceivable new virulence phenomenon in aEPEC. However, it has seldom been studied how these or other aEPEC strains interact with the mucus layer or whether they can break and use mucin to grow. In addition, the mechanisms used by aEPEC strains to induce mucus hyperproduction *in vivo* or *in vitro* are unknown. To clarify some of these topics, we evaluated different aspects related to the mucus interaction of thirteen aEPEC strains and compared the genome of the mucus-inducers with those that could induce mucus production.

After an extensive evaluation of the genome of the 13 aEPEC strains in this study, we verified that the two strains known to induce mucus hyperproduction (0421-1 and 3991-1) (Vieira et al., 2010), were grouped in the same phylogroup (A), intimin subtype (iota 2), ST (378) and serotype (O101/O162:H33). The close phylogenetic relationships between these strains were only recognized after genome sequencing since they initially displayed different serotypes. The EPEC pathotype is commonly related to phylogroup A and B1 (Hernandes et al., 2020). Moreover, phylogroup A has been detected in aEPEC ancestry analysis studies (Afset et al., 2008; Bando et al., 2009), highlighting the extensive phylogenetic diversity within this group. However, the ST378 is not commonly reported in the literature, suggesting that only a limited number of isolates from this group have undergone a comprehensive study.

Interestingly, two other strains from the aEPEC collection (1582-4 and 2531-13), isolated from different patients on different dates, were found to have a close genetic relationship with strains 0421-1 and 3991-1. In addition, in this study, we showed that they shared the ability to induce mucus hyperproduction in the rabbit ileal loop model. Notably, among the strains studied in the present work, seven belonged to the global lineage EPEC10 and were distributed into two different clusters. However, only the strains clustered with the 0421-1 and 3991-1 strains were found to induce mucus production, while the cluster composed of strains 1711-4, 4361-1, and 4581-2 did not. Therefore, the other four strains from ST378 that were isolated in different countries and clustered together with the 0421-1 and 3991-1 strains may also have the ability to induce mucus production.

Previous studies have already demonstrated that some intimin subtypes are associated with specific phenotypes, such as the omicron subtype being associated with the cellular invasion phenotype (Pacheco et al., 2014). Our results showed that the four mucus-inducing strains shared the intimin ι2 (iota two) subtype, which is not frequent in strains isolated from human feces (Zhang et al., 2002). This interesting finding may serve as a basis for future investigations into the potential involvement of specific intimin subtypes with the induction of mucus production or even the role of other components related to the T3SS or the T3SS-dependent effectors.

The genome analysis of the aEPEC strains in our study revealed the presence of multiple VFs and a high level of diversity in the combination of these factors, supporting previous studies that highlighted the genetic diversity of the aEPEC pathotype (Afset et al., 2008; Bando et al., 2009; Hernandes et al., 2009, 2020). Although the four mucus-inducing strains (0421-1, 1582-4, 2531-13, and 3991-1) were genetically closely related, they only shared 22 known VFs out of the 75 genes found in at least one strain. It is worth noting that these VFs were also present in the other aEPEC strains, including those that could not induce mucus production.

LEE and non-LEE effectors play a crucial role in the pathogenicity of aEPEC. These effectors can remodel a cell’s cytoskeleton and modulate the immune system (Wu et al., 2010; Gao et al., 2013; Pearson et al., 2013; Ingle et al., 2016; Cepeda-Molero et al., 2020). Among the 40 known effectors secreted by the T3SS, none were exclusive to the mucus-inducing strains. Typically, aEPEC strains carry the pathogenicity island (PAI) LEE and produce a T3SS (Nataro and Kaper, 1998; Trabulsi et al., 2002; Kaper et al., 2004), which means that they are expected to have the effectors and components of this system (EspA, EspB, EspF, EspG, EspH, EspZ, Tir, and Map). The Nle effectors have various functions, including inhibiting the NF-κB cellular signaling pathway and host cell apoptosis (Pearson et al., 2013; Ingle et al., 2016). The four strains of aEPEC under study have a single non-LEE effector in common, NleB2-1. This effector is involved in inhibiting the activation of the NF-κB pathway, modulating inflammatory pathways, and regulating cell survival or apoptosis, acting as a cell death signaling blocker (Gao et al., 2013; Cepeda-Molero et al., 2020). Therefore, pro-inflammatory pathways may not be associated with the induction of mucus hypersecretion in infected patients.

A pangenome analysis was performed to compare the strains that induce mucus production (0421-1, 1582-4, 2531-1, and 3991-1) with those that do not (1711-4, 3391-3, 3881-3, and 4361-1 4581-2). However, no specific gene or set of genes related to the strains that induce mucus production was identified. While 252 genes were found exclusively in the mucus-inducing strains, most encoded were hypothetical proteins and require further characterization to determine their role in the mucus-inducing phenotype.

Although mucus production and consumption are independent events, Vieira et al. (2010) found that the aEPEC 3991-1 strain induces mucus production, which it uses as a carbon source to promote its growth. Therefore, the induction of mucus production and its degradation could be linked as an advantage to overcome other bacteria in the gut, and perhaps the degradation process could be related to mucus production. For example, the autotransporter protein Pic has a dual function: it acts as a mucus secretagogue and a mucinase, contributing to bacterial nutrition and penetration of the mucus layer to access epithelial cells (Flores-Sanchez et al., 2020).

When mucinase-encoding genes were screened in the genomes of the strains, only SslE was identified in three of them. The SslE protein helps the bacteria penetrate the mucus layer to reach the underlying epithelial cells (Nesta et al., 2014; Valeri et al., 2015), which may be necessary in some aEPEC pathogenesis. However, it cannot be linked to the induction of mucus production as none of the mucus-inducing strains carried the SslE operon. It is worth noting that the genome of the 3991-1 strain and other mucus inducers did not contain any known mucinase-encoding genes. However, all of them used mucus as a carbon source, indicating that other enzyme-encoding genes, such as a non-characterized mucinase gene, might be present in the genome of these strains.

Three different *in vitro* methods were used to investigate the mucinolytic activity of aEPEC. The results showed that the aEPEC collection studied could interact with, penetrate, and utilize porcine gastric mucin. The mucus layer is the first line of defense against pathogenic bacteria and provides various growth substrates, primarily in O-glycosylated mucins. These mucins are highly polymorphic and can mediate specific binding of microorganisms along the gastrointestinal tract. To investigate the binding of aEPEC to mucin, we plated the bacteria onto mucin agar plates, which had been previously used to assess the specificity of *B. cereus* and ETEC strains to mucins (Tsilia et al., 2015; Sauvaitre et al., 2022). Only four aEPEC strains (70, 1711-4, 2731-1, and 4581-2) showed enhanced adhesion compared to agar plates devoid of mucin. Therefore, it is crucial to use both binding and degradation assays to determine the mucinolytic potential of a pathogenic strain. These findings highlight the importance of conducting both tests to understand the virulence of the strains under investigation.

The binding sites on the surface of mucin are diverse and similar to those found in epithelial cells. Since the outer mucus layer is constantly produced and removed with a short lifespan, this positive interaction can hinder the pathogenicity of harmful microorganisms by preventing their colonization and translocation to the underlying epithelial cells (Juge, 2019).

On the other hand, mucus-binding also may contribute to improving the bacterial anchorage on the adherent inner mucus layer. Depending on its location in the lumen and thickness, bacteria may breach this barrier by degrading these oligomeric mucins or influencing a constant secretion to support their colonization (Rolhion and Chassaing, 2016). Even though mucin degradation seems to depend on specific mucinases, this still needs to be demonstrated. A recent study has shown that mucin degradation involves multiple bacterial enzymes, such as proteases, glycosidases, and sulfatases (Drula et al., 2022). All these enzymes are essential to facilitate bacterial penetration and progression into the epithelium. Moreover, the complete degradation of mucin requires the combined action of both commensal and pathogenic bacteria (Hoskins and Boulding, 1981; Corfield et al., 1992).

As part of our study, we examined mucin degradation over a short-term incubation period using a minimal medium where mucin was the only carbon source. We found that all aEPEC strains displayed a significant growth rate in a mucin medium, indicating their potential to metabolize this complex substrate. Additionally, we evaluated the ability of aEPEC to penetrate the mucus barrier, likely due to breaking down mucin. Interestingly, not all aEPEC strains could break down the dense mucus layer (1582-4, 2531-13, 3391-3, and MA3456). We hypothesize that their behavior may be due to a slower growth rate or because the components of the DMEM medium, which is located above the mucus barrier in the Transwell, offer better metabolic resources than the oligosaccharide chains present along the mucin surface. In addition, we found that five strains (51, 70, 0421-1, 1711-4, and 3881-3) crossed the barrier much more efficiently than the positive controls, while two other strains (2731-1 and 3991-1) were able to cross it significantly less. However, it is important to note that this behavior cannot be attributed to any strain’s ability to induce mucus production.

We highlight the hypothesis that the bacterial flagellum activity may enhance the ability to penetrate the mucus layer since most strains capable of penetrating more than the positive control (EAEC strain 042) are motile. Non-motile strains (2731-1, 3991-1, and 2012-1) penetrated the mucus layer less significantly than 042. The bacterial flagellum plays a significant role in the mobility of bacteria in cervical mucus, enabling the bacteria to change direction quickly over a short period (Figueroa-Morales et al., 2019). The two mucus-inducing strains (3991-1 and 0421-1) penetrated the mucus layer, and the motile strain (0421-1) penetrated significantly more than the non-motile (3991-1). To confirm the hypothesis of the importance of the flagellum, we evaluated strain 1711-4 and its non-motile isogenic 1711-4 Δ*fliC* (deleted in flagellin) mutant. Both could penetrate the mucus layer, but the motile isogenic strain penetrated the mucus layer significantly (not shown), confirming that bacterial motility improves penetration.

Although commercial porcine mucin mainly contains aggregates of MUC5AC type, which is a secreted gel-forming glycoprotein produced in the stomach epithelium, it differs in size from human MUC2 intestinal mucin due to its large number of O-glycosylated chains with specific domains that facilitate host-pathogen interaction. However, it is widely used as an experimental substitute since both share similar properties (Labourel et al., 2023). Its composition is well-described and contains N-acetylglucosamine (GlcNac), N-acetylgalactosamine (GalNac), galactose, fucose, glucose, sialic acids, sulfate, and proteins, which allows for a more accurate investigation of mucin degradation by commensal and pathogenic microorganisms. Additionally, crude extracts from human mucin are limited and less available (Marczynski et al., 2021).

Our findings indicate that the *in vivo* mucus induction by aEPEC strains is not attributable to a single genetic element. No unique virulence factor or gene set was identified exclusively in the mucus-inducing strains, nor was there a factor present in non-inducing strains and absent in the inducing ones, suggesting the multifactorial nature of this phenomenon. The molecular mechanisms responsible for the observed differences in cellular interaction and response and the relationship between VFs and mucus induction in aEPEC-infected cells require further investigation. However, we emphasize that, due to the limited research available in the literature on mucus induction in intestinal cells by aEPEC infection, our work stands out as both innovative and pioneering, providing a solid basis for future studies in the area.

## 5 Conclusions

aEPEC is a highly diverse group of pathogens with broad genetic, molecular, and phenotypic plasticity. Perhaps the most significant obstacle to molecular investigations of aEPEC is the heterogeneity of genetic markers, which often makes it difficult to extrapolate the results of one isolate to another. This feature makes it more challenging to search for a determining factor that helps to elucidate the phenomenon of *in vivo* mucus induction, which may be more common among aEPEC strains than expected. Our results also provide convincing evidence of the mucinolytic potential from a diverse group of aEPEC strains. More in-depth studies are still needed, covering phenotypic and molecular aspects that trace a path directed toward understanding the specific mechanism of intestinal mucus colonization.

## Data Availability

The eight *E. coli* strain sequenced and published in the present work have been deposited at DDBJ/ENA/GenBank under the Bioproject number PRJNA872550; the accession number for the thirteen strains used in the present work can be found in **Table 3**.
