# Supplementary material for: Identification of a genomic cluster related to hypersecretion of intestinal mucus and mucinolytic activity of atypical enteropathogenic Escherichia coli (aEPEC)
Source: Front Cell Infect Microbiol. 2024 Dec 4;14:1393369. doi: 10.3389/fcimb.2024.1393369 (PMC11656320; doi:10.3389/fcimb.2024.1393369)
Supplement: Supplementary file 3 [file Table3.docx]

Table S3 – Information regarding genome sequencing metric obtained for the eight aEPEC strains sequenced in the present study

| Strain | Number of reads with inserts >300 bp (n) | Mean of insert size (bp) | Mean coverage (×) |
| --- | --- | --- | --- |
| 51 | 1381856 | 626 | 140.775 |
| 70 | 976523 | 625 | 101.394 |
| 0421-1 | 929211 | 688 | 102.716 |
| 1582-4 | 910896 | 679 | 96.8236 |
| 2731-1 | 850245 | 653 | 89.2038 |
| 3391-3 | 1149933 | 644 | 109.954 |
| 3991-1 | 1161287 | 643 | 129.34 |
| 4361-1 | 852200 | 656 | 94.7663 |
